# UK DRAFFT - A randomised controlled trial of percutaneous fixation with kirschner wires versus volar locking-plate fixation in the treatment of adult patients with a dorsally displaced fracture of the distal radius

**DOI:** 10.1186/1471-2474-12-201

**Published:** 2011-09-13

**Authors:** Matthew L Costa, Juul Achten, Nick R Parsons, Amar Rangan, Richard P Edlin, Jaclyn Brown, Sarah E Lamb

**Affiliations:** 1 Division of Health Sciences, Warwick Medical School, The University of Warwick, Clifford bridge road, Coventry, CV2 2DX, UK; 2 Wolfson Research Institute, School of Medicine & Health, Durham University, Queen's Campus, University Boulevard, Teesside TS17 6BH, UK; 3 Leeds Institute of Health Sciences, Leeds University, Charles Thackrah Building, 101 Clarendon Road, Leeds, LS2 9LJ, UK; 4 Clinical Trials Unit, Warwick Medical School, The University of Warwick, Gibbet Hill Campus, Coventry, CV4 7AL, UK

## Abstract

**Background:**

Fractures of the distal radius are extremely common injuries in adults. However, the optimal management remains controversial. In general, fractures of the distal radius are treated non-operatively if the bone fragments can be held in anatomical alignment by a plaster cast or orthotic. However, if this is not possible, then operative fixation is required. There are several operative options but the two most common in the UK, are Kirschner-wire fixation (K-wires) and volar plate fixation using fixed-angle screws (locking-plates). The primary aim of this trial is to determine if there is a difference in the Patient-Reported Wrist Evaluation one year following K-wire fixation versus locking-plate fixation for adult patients with a dorsally-displaced fracture of the distal radius.

**Methods/design:**

All adult patients with an acute, dorsally-displaced fracture of the distal radius, requiring operative fixation are potentially eligible to take part in this study. A total of 390 consenting patients will be randomly allocated to either K-wire fixation or locking-plate fixation. The surgery will be performed in trauma units across the UK using the preferred technique of the treating surgeon. Data regarding wrist function, quality of life, complications and costs will be collected at six weeks and three, six and twelve months following the injury. The primary outcome measure will be wrist function with a parallel economic analysis.

**Discussion:**

This pragmatic, multi-centre trial is due to deliver results in December 2013.

**Trial registration:**

Current Controlled Trials ISRCTN31379280

UKCRN portfolio ID 8956

## Background

Fractures of the distal radius are extremely common injuries. In the Western World, 6% of women will have sustained such a fracture by the age of 80 and 9% by the age of 90 [1]. The optimal management of fractures of the distal radius in adults remains controversial. There is a bimodal distribution in terms of age. Younger patients frequently sustain complicated, high-energy injuries involving the wrist joint. However, fractures of the distal radius are also common in older patients who are more likely to sustain low-energy fractures, often related to osteoporosis [2] This study is designed to address both groups of patients as the key management issues pertain to all patients with a fracture of the distal radius.

In general, fractures of the distal radius are treated non-operatively if the bone fragments are undisplaced or the fragments can be held in anatomical alignment (reduction) by a plaster cast or orthotic. However, if this is not possible then operative fixation is required. This carries inherent risks for the patient and considerable cost implications for the NHS; much of this cost is related to the choice of fixation [3].

There are several operative options but the two most common in the UK, are Kirschner-wire fixation (K-wires) and volar plate fixation using fixed-angle screws (locking-plates). Each surgical method has its own advantages and disadvantages:

K-wire fixation is a long-standing and widely practised technique. During this procedure smooth metal wires with a sharp point are passed across the fracture site through the skin. This is a relatively simple, quick, and minimally invasive technique, which is cheap and requires limited operative hardware. However, since the fixation is not 'rigid' (the wires are inherently flexible) the wrist has to be immobilised in plaster cast; normally for six weeks or until the wires are removed. There is a risk of infection where the wires enter the skin. There is also a risk that the fracture will 'collapse' when the wires are removed, leading to deformity and loss of function [4].

Locking-plate fixation, in the distal radius and for other fractures, has been facilitated by recent advances in implant technology which allow the screws to be 'locked' into the plate. This produces a 'fixed-angle' bone-plate construct, (previously, plate-and-screw constructs relied on friction alone to maintain their position on the bone). Although originally designed for use in osteoporotic bone specifically, the theoretical advantages of the locking-plates may equally be applied to high-energy (often multi-fragmentary) fractures in younger patients. The technique has become increasingly popular in both the UK and across the developed world over the last five years. The procedure requires an incision over the volar (palm) side of the wrist. The plate and screws are then applied to the bone fragments under direct vision. This produces a rigid construct, [5] and therefore the patients can be permitted to mobilise their wrist more quickly, potentially reducing future stiffness. Since the plate and screws can remain inside the patient permanently, the risk of later collapse of the fracture is also smaller. However, this technique takes longer than a K-wire fixation and there is a risk of serious intra-operative complications such as injury to a nerve or blood vessel [5]. There is also a risk of flexor and/or extensor tendon irritation and rupture [6]. The locking-plate hardware itself is specialised and considerably more expensive.

In 2003, Handoll and Madhok [7] summarised the results of a series of Cochrane Reviews of randomised controlled trials of the treatment of fractures of the distal radius and "exposed the serious deficiency in the available evidence". However, they were able to identify key areas for future research including "when and what type of surgery is indicated".

The null hypothesis for this trial is that there is no difference in the Patient-reported Wrist Evaluation following K-wire fixation versus 'locking-plate' fixation for patients with a dorsally-displaced fracture of the distal radius.

## Methods/Design

### Design

This is a pragmatic, multi-centre, randomised clinical trial with parallel economic analysis. The study was approved by the Research Ethic Committee (Ref: 10/H1210/10) and the NHS CSP (Ref:NIHR CRN study ID 8956), and registered with the International Standard Randomised Controlled Trial Register (Ref: ISRCTN31379280).

### Study participants

Patients will be eligible for this study if:

• They have sustained a dorsally displaced fracture of the distal radius, which is defined as a fracture within 3 cm of the radio-carpal joint.

• The treating surgeon believes that they would benefit from operative fixation of the fracture.

• They are over the age of 18 and able to give informed consent.

• The patient presents within two weeks of the injury

Patients will be excluded from participation in this study if:

• The fracture extends more than 3 cm from radio carpal joint

• The fracture is open with a Gustillo grading greater than 1

• The articular surface of the fracture cannot be reduced by indirect techniques. (In a small number of fractures, the joint surface is so badly disrupted that the surgeon will have to open up the fracture in order to restore the anatomy under 'direct' vision).

• There are contra-indications to anaesthetic.

• There is evidence that the patient would be unable to adhere to trial procedures or complete questionnaires, such as cognitive impairment or intravenous drug abuse

### Trial Interventions

All of the hospitals involved in this trial currently use both of the methods of fixation and all of the surgeons involved will be familiar with both techniques. Operative fixation of fractures of the distal radius usually takes place under a general anaesthetic but this decision will be made by the attending anaesthetist. Each patient will undergo the allocated surgery according to the preferred technique of the operating surgeon. Although, the basic principles of K-wire fixation and locking-plate fixation are inherent in the technique, there are several different implant systems and several different options for the positioning of wires and screws. In this trial, the details of the surgery will be left entirely to the discretion of the surgeon to ensure that the results of the trial can be generalised to as wide a group of patients as possible.

#### K-wire Fixation

The wires are passed through the skin over the dorsal aspect of the distal radius and into the bone in order to hold the fracture in the correct (anatomical) position. The size and number of wires, the insertion technique and the configuration of wires will be left entirely to the discretion of the surgeon. A plaster cast will be applied at the end of the procedure to supplement the wire fixation as per standard surgical practice. This cast holds the wrist still and is left on until the wires are removed at the 6-week follow-up appointment.

#### Locking-plate Fixation

The locking-plate is applied through an incision over the volar (palm) aspect of the wrist. Again, the details of the surgical approach, the type of plate, and the number and configuration of screws will be left to the discretion of the surgeon. The screws in the distal portion of the bone will be 'fixed-angle' i.e. screwed into the plate, but this is standard technique for the use of these plates. The type of proximal screw will be left to the discretion of the surgeon; these may be locking or non-locking screws as the bone in this area provides a much better purchase for the screws. Some surgeons use a temporary plaster cast to hold the patients' wrist still but the fixed-angle stability provided by the locking-plate is generally sufficient to allow early controlled range-of-movement exercises. The use or otherwise of a cast will again be left to the discretion of the surgeon as per usual practice.

#### Rehabilitation

Patients randomised into the two groups will receive standardised, written physiotherapy advice detailing the exercises they need to perform for rehabilitation following their injury. All of the patients in both groups will be advised to move their shoulder, elbow and finger joints fully within the limits of their comfort. Those patients in the K-wire group will be encouraged to perform range-of-movement exercises at the wrist as soon as their plaster cast is removed at the follow-up appointment. Those patients in the locking-plate group may begin the exercises immediately if they do not have a plaster cast or as soon as the cast is removed. In this pragmatic trial, any other rehabilitation input beyond the written information sheet (including a formal referral to physiotherapy) will be left to the discretion of the treating surgeon. However, a record of any additional rehabilitation input (type of input and number of additional appointments) together with a record of any other investigations/interventions will be requested as part of the 3 month, 6 month and 12 month postal follow-ups and this will also form part of the trial dataset.

### Outcome Measures

Patient characteristics and baseline (pre-injury) functional status will be collected after consent to take part in the trial. Structured information regarding other injuries which may affect outcome e.g. disruption of the carpal ligaments, will be collected but all patients will be included in the analysis.

The primary outcome measure for this study is the ***Patient Rated Wrist Evaluation ***[8]. The PRWE score is a validated questionnaire which is self-reported. It consists of 15 items specifically related to the function of the wrist. This data will be collected at baseline, 3, 6 and 12 months post-operatively. The PRWE is the most sensitive outcome measure for patients sustaining this specific injury [9].

The secondary outcome measures in this trial are: the ***Disabilities of Arm, Shoulder and Hand score***; The DASH Outcome Measure is a 30-item, self-report questionnaire designed to provide a more general measure of physical function and symptoms in people with musculoskeletal disorders of the upper limb [10], ***EQ-5D***; The EQ-5D is a validated, generalised, quality of life questionnaire consisting of 5 domains related to daily activities with a 3-level answer possibility. The combination of answers leads to the QoL score [11], ***Complications***; all complications will be recorded. **Radiographic evaluation**; Standard posterior-anterior and lateral radiographs will be taken at baseline, 6-weeks and 12 months after the injury, ***Resource use ***will be monitored for the economic analysis. Unit cost data will be obtained from national databases such as NHS Reference costs, the BNF and PSSRU Costs of Health and Social Care [12]. Where these are not available the unit cost will be estimated in consultation with the finance department at the lead hospital. The cost consequences following discharge, including NHS costs and patients' out-of-pocket expenses will be recorded via a short questionnaire which will be administered at 3, 6 and 12 months post surgery. Patient self-reported information on service use has been shown to be accurate in terms of the intensity of use of different services [13].

We will use techniques common in long-term cohort studies to ensure minimum loss to follow-up, such as collection of multiple contact addresses and telephone numbers, mobile telephone numbers and email addresses. Considerable efforts will be made by the trial team to keep in touch with patients throughout the trial by means of newsletters etc.

### Sample size

The Patient Rated Wrist Evaluation (PRWE) score [8] is 15-item questionnaire, that rates wrist function using a range of questions in two (equally weighted) sections concerning the patient's experience of pain and disability. Scoring for all the questions is via a 10-point, ordered, categorical scale ranging from 'no pain' or 'no difficult' (0) to 'worst possible pain' or 'unable to do' (10). Five questions relate to a patient's experience of pain and ten relate to function and disability; scores for the ten function items are summed and divided by two and added to the five pain items to give a score out of 100 (best score = 0 and worst score = 100).

A 6-point difference between groups at the 5% level with 80% power requires 175 patients in each group (*Power and sample size software, available at *http://biostat.mc.vanderbilt.edu/wiki/Main/PowerSampleSize). A 6-point difference between groups equates to a standardized effect size of 0.3, for an assumed standard deviation of 20 points [14]. MacDermid et al [9], found that the PRWE is sensitive enough to detect subtle but clinically relevant changes in wrist function of this order of magnitude in patients sustaining a fracture of the distal radius; for example changes between 3 and 6 months. At the individual level, a change in the PRWE of 6 points reflects the difference between turning a doorknob or cutting a loaf of bread with mild pain versus no pain. We believe that such an improvement is important to patients on an individual and population level and could lead to a change in clinical practice in the UK.

In summary, this study will use the **PRWE score at 12 months after surgery **as the primary outcome measure. The total number of patients required to obtain a power of 80% to detect a 6-point difference between groups for the primary outcome measure will be 350; i.e. 175 patients will be required in each treatment group. In trials run previously at our institution comparing two different surgical techniques we experienced a ~5% loss to follow-up. With an allowance for a conservative 10% loss to follow-up, we would plan to recruit **390 patients **in total.

### Randomisation

After patients have provided baseline assessments and been checked for eligibility they will be asked for their informed consent to take part in the trial. The method of fixation will be allocated using a secure, centralised, telephone randomisation service. Randomisation will be on a 1:1 basis, stratified by centre, intra-articular extension of the fracture and age of the patient (above or below 50 years):

*Stratification by centre *will help to ensure that any clustering effect related to the centre itself will be equally distributed in the trial arms.

*Stratification on the basis of intra-articular extension of the fracture *(specifically involvement of the articular surface of the radio-carpal joint) will eliminate a major potential confounder, since disruption of this articular surface may pre-dispose to secondary osteoarthritis of the wrist [15].

*Stratification on the basis of age *will be used to discriminate between younger patients with normal bone quality sustaining high-energy fractures, and older patients with low-energy (fragility) fractures related to osteoporosis. Age will therefore be used as a surrogate for bone density. In a large study in Norway involving 7600 participants, it was demonstrated that forearm bone mineral density remains stable up until the age of 50 years. After the age of 50, bone mineral density decreased steadily in males, whilst in females there was an initial decline between the ages of 50 and 65, with a further decline in the age groups thereafter [16].

### Statistical Analysis

Missing data are not expected to be a problem for this study. However, if appropriate, missing data will be imputed using the multiple imputation facilities (mice package) available in R (http://www.r-project.org/). If the degree of missingness is relatively low, as expected, the primary analysis will be based on complete cases only (*complete case analysis*), with analysis of imputed datasets used to assess the sensitivity of the analysis to the missing data.

Standard statistical summaries (e.g. medians and ranges or means and variances, dependent on the distribution of the outcome) and graphical plots showing correlations will be presented for the primary outcome measure and all secondary outcome measures. Baseline data (e.g. age and gender) will be summarized to check comparability between treatment arms, and to highlight any characteristic differences between those individuals in the study, those ineligible, and those eligible but withholding consent.

Differences between treatment groups will be assessed on an intention-to-treat basis, using on a normal approximation for the PRWE score, at 12 months post-operatively, and at interim occasions. Tests will be two-sided and considered to provide evidence for a significant difference if p-values are less than 0.05 (5% significance level). Estimates of treatment effects will be presented with 95% confidence intervals. A multi-level modeling approach, where patients are naturally grouped by surgeons, and likewise surgeons are grouped by recruiting centres will be used for the primary analysis. This model will formally incorporate terms that allow for possible heterogeneity in responses for patients due to the recruiting centre and the surgeon, in addition to the fixed effects of the treatment groups, patient age and intra-articular extension. Although we expect, given that an individual surgeon will only operate on a small number of patients, that actually a simpler model consisting of a single random effect accounting for the recruiting centre will be used. The main analyses will be conducted using specialist multi-level modeling functions available in the software package R, where PRWE data are assumed to be normally distributed, possibly after appropriate variance-stabilising transformation.

The temporal patterns of any complications will be presented graphically and if appropriate a time-to-event analysis (Kaplan-Meier survival analysis) will be used to assess the overall risk and risk within individual classes of complications (e.g. infection).

The statistical analysis plan (SAP) will be agreed with the Data Management Committee (DMC) at the start of the study. Any subsequent amendments to this initial SAP will be clearly stated and justified. Interim analyses will be performed only where directed by the DMC. The routine statistical analysis will mainly be carried out using R and S-PLUS (http://spotfire.tibco.com/products/s-plus/statistical-analysis-software.aspx).

### Health Economics Analysis

The economic evaluation will estimate costs of both treatments, and if appropriate the incremental cost effectiveness of distal radial fractures treated by locking-plate fixation versus K-wire fixation for 1) patients under 50 years of age and 2) patients over 50 years of age. The primary outcome for the economic evaluation will be the Quality Adjusted Life Year gained. Health related quality of life will be estimated using the EuroQol (EQ-5D). This data will be collected at baseline (pre-injury and immediate post-injury) 3, 6 and 12 months post operatively. Hospital based resource use will be extracted from patient records. Primary, community and social care service usage will be collected using a patient questionnaire, at 3, 6 and 12 months. Patients will also have the opportunity to detail out of pocket expenditure related to their treatment in the diary. Unit cost data will be obtained from national databases such as NHS reference costs, the BNF and PSSRU Costs of Health and Social Care. Where these are not available the unit cost will be estimated in consultation with the finance officer in the lead hospital department.

Two economic evaluations will be undertaken for each age group. A within trial evaluation will compare the outcomes and cost up to 12 months follow-up using trial data. A longer term evaluation will model outcomes and costs up 10 years post-surgery. For both analyses the perspective will be that of the UK NHS and Social Services. The discount rate will be 3.5% as per the NICE Methods Guide and parameter uncertainty will be addressed through probabilistic sensitivity analysis. Outputs of the analyses will be presented as expected incremental cost effectiveness ratios, cost effectiveness acceptability curves/frontiers, plus expected net benefit assuming a threshold value of £30,000 per QALY in line with the NICE reference case [17].

## Discussion

This pragmatic, multi-centre trial is due to deliver results in December 2013.

## Abbreviations

BNF: British National Formula; DASH: Disabilities of Arm, Shoulder and Hand; DMC: Data Management Committee; NICE: National Institute for Health and Clinical Excellence; PSSRU: Personal Social Services Research Unit; PRWE: Patient reported Wrist Evaluation; QALY: Quality Adjusted Life Year; SAP: Statistical Analysis Plan.

## Competing interests

The authors declare that they have no competing interests.

## Authors' contributions

MC developed the protocol and has overall clinical responsibility for the conduct of the trial. AR developed the protocol and is responsible for recruitment of patients. JA developed the protocol and is a member of the trial management group. JB developed the protocol and is the trial manager. NP developed and is responsible for the statistical analysis of the trial. RE developed and is responsible for the health economics analysis. SL developed the protocol and is a member of the trial management group. All authors read and approved the final manuscript.

## Pre-publication history

The pre-publication history for this paper can be accessed here:

http://www.biomedcentral.com/1471-2474/12/201/prepub
